# The rete mirabile: a possible control site for swimbladder function

**DOI:** 10.1007/s00360-023-01486-5

**Published:** 2023-04-15

**Authors:** Bernd Pelster

**Affiliations:** 1 Institute for Zoology, Leopold-Franzens-Universität Innsbruck, Technikerstr. 25, 6020 Innsbruck, Austria; 2 Center for Molecular Biosciences, Universität Innsbruck, Innsbruck, Austria

**Keywords:** Rete mirabile, Swimbladder, European eel, Blood flow control, Receptor, Intracellular signaling

## Abstract

In a recent study, a large number of transport proteins was detected in the transcriptome and proteome of saline perfused rete mirabile tissue of the European eel. In this study, the data set was reanalyzed for the presence of receptor proteins and proteins involved in intracellular signaling pathways. A large number of expressed receptor proteins and proteins involved in intracellular signal transduction was detected. Several G-protein-coupled receptor signal pathways were significantly enriched in their expression level, in particular receptors and signaling pathways involved in the control of blood flow. The enriched signaling pathways also include pathways involved in trafficking of crucial transport proteins like, monocarboxylate transporters, V-ATPase, and aquaporin. The data, therefore, suggest that the rete mirabile has the capacity to control swimbladder function by regulating blood flow and by modifying countercurrent multiplication.

**Supplementary Information:**

The online version contains supplementary material available at 10.1007/s00360-023-01486-5.

## Introduction

Countercurrent systems are widespread in the animal kingdom and used to concentrate heat or gases (Stevens 2011). Countercurrent heat exchange allows for the concentration of heat in the body core, while peripheral tissues stay cold, as observed, for example, in some fish with elevated body temperatures, in some birds or in mammals (Scholander and Schevill 1955; Dickson and Graham 2004; Stevens 2011; McCafferty et al. 2013). It may also be used for selective brain cooling (Jessen 2001; Strauss et al. 2017). In fish, a countercurrent system is used for the concentration of gases in the eye and in the swimbladder. In the choroid rete of the eye oxygen is concentrated by countercurrent concentration to supply the avascular retina (Pelster 2001; Berenbrink et al. 2005; Waser 2011). In physoclist fish a countercurrent system, the rete mirabile, is used to concentrate gases for the filling of the swimbladder under elevated hydrostatic pressure. Presence of fish with gas filled swimbladder at a depth of several hundred or even more than 1000 m demonstrates that extremely high gas partial pressures can be generated (Marshall 1960; Pelster 1997, 2001, 2023).

The European eel is physostome, but the connection between the esophagus and the swimbladder is closed soon after opening the swimbladder in the glass eel stage, therefore, the eel is functionally physoclist (Dorn 1961; Zwerger et al. 2002). While in many fish the rete mirabile is intimately connected to gas gland cells of the swimbladder, in the eel the rete mirabile is bipolar, allowing access to larger blood vessels immediately in front and behind the rete, and the eel thus became a model for the analysis of swimbladder function (Steen 1963; Fänge 1983; Pelster 2001).

In the European eel rete mirabile about 58.000 arterial capillaries are surrounded by about 44.000 venous capillaries (Krogh 1929) for a distance of a few millimeters, and the diffusion distance between arterial and venous vessels is in the range of only 1–2 µm (Stray-Pedersen and Nicolaysen 1975; Wagner et al. 1987). Thus, the rete provides a large surface area with very short diffusion distance for the back-diffusion of gases. Blood samples collected in front of and immediately behind the rete of a European eel swimbladder preparation revealed a seven- to eightfold increase in PO_2_ and PCO_2_ during arterial passage of the rete, resulting from back-diffusion and countercurrent concentration in the rete (Kobayashi et al. 1990).

Retia mirabilia are typically considered to allow for the countercurrent concentration of gases or of heat (Kuhn et al. 1963; Stevens 2011). In the European eel measurements of lactate, water and hemoglobin concentration, however, indicated that lactate may also be concentrated in the rete by back-diffusion from venous to arterial capillaries (Kobayashi et al. 1989a). A recent study of the transcriptome and the proteome of rete mirabile tissue of the European eel indeed demonstrated the presence of a large number of transport proteins and membrane ATPases (Schneebauer et al. 2021). Membrane transport proteins, however, are not always constitutively active, their activity may be adjusted in relation to cellular activity. This appears especially important for the rete as model calculations revealed that countercurrent concentration of lactate in the rete mirabile would enhance countercurrent concentration of gases (Kobayashi et al. 1989b). The presence of membrane transport proteins in rete mirabile membranes with adjustable activity thus could significantly affect the gas concentrating ability of the rete. Moreover, some receptor proteins have been detected in the study of Schneebauer et al. (Schneebauer et al. 2021), and receptor proteins require intracellular signaling cascades, but this was not in the focus of that study. This study, therefore, was set out to reanalyze the transcriptome and proteome data published by Schneebauer et al. (Schneebauer et al. 2021) searching in particular for receptor proteins and proteins involved in intracellular signaling. The results revealed expression of a large number of receptor proteins, and of several intracellular signaling pathways, connected to these receptors. The results provide evidence that blood flow through the swimbladder may be controlled at the level of the rete mirabile, and signaling pathways involved in the insertion of crucial transport proteins into the cell membranes are enriched in their expression level.

## Methods

For this study, transcriptome (accession number GSE172092, https://www.ncbi.nlm.nih.gov/geo/query/acc.cgi?acc=GSE172092) and proteome data (accession number PXD025435, https://www.ebi.ac.uk/pride/archive/projects/PXD025435), published by Schneebauer et al. (2021), were reanalyzed focusing on the presence of transcripts coding for receptor proteins and for proteins connected to intracellular signaling, and on the presence of the appropriate proteins. Transcripts and proteins were selected by searching the ‘*Description*’ and the ‘*GO biological function*’ of annotated transcripts and proteins for the terms ‘receptor’ and ‘signaling’. Extracted transcripts and proteins were then used to identify signaling pathways that are expressed more than would be expected by chance in rete tissue by performing a GO enrichment analysis using the GO Ontology database https://doi.org/10.5281/zenodo.6799722, released 2022-07-01. Based on this enrichment analysis signaling pathways were identified that may be of functional importance for rete tissue. GO pathways are defined based on mammalian data, but meanwhile a large number of non-mammalian species have been added, including the zebrafish, *Danio rerio* (see http://geneontology.org/), and a remarkable number of orthologue genes and of overlap has been detected between different vertebrates, including mammals and fish (Howe et al. 2013).

## Results

The description of identified transcripts of rete mirabile tissue revealed presence of 1808 mRNA transcripts coding for a receptor protein. Searching the GO biological function of identified transcripts for the term ‘signaling’ identified 7206 transcripts. Analyzing the proteome for the same terms revealed presence of 136 receptor proteins and 1291 proteins connected to the biological function ‘signaling’. 299 of these proteins were detected in the transcriptome as well as in the proteome (Suppl. Tab. 1).

**Fig. 1:**
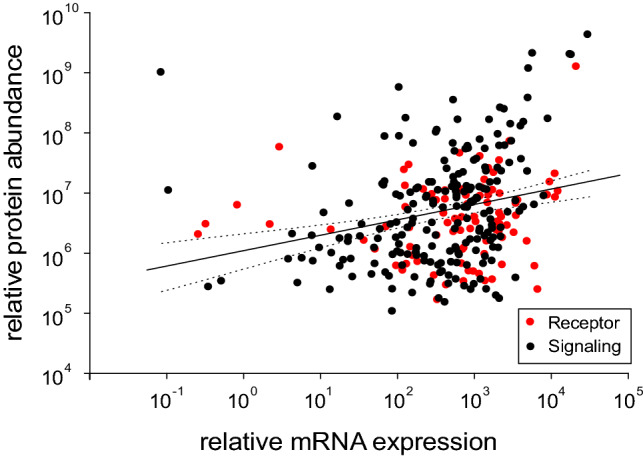
Relative mRNA expression plotted versus the relative protein abundance of genes coding for a receptor protein or a protein connected to intracellular signal transduction detected in the proteome as well as in the transcriptome of European eel rete mirabile tissue. Regression line intercept: 10^6.02^ (0.15); log 10(×) 0.25 (0.05); *R*^2^ 0.07; *p* < 0.001; calculated with ‘*R*’. Broken lines show 95% confidence interval

In Fig. 1 for all genes detected in the transcriptome as well as in the proteome the relative mRNA expression value is plotted versus the relative protein abundance. Relative mRNA expression values usually were between 10^0^ and 10^5^, while the relative protein abundance covered the range between 10^5^ and 10^10^.

The GO enrichment analysis confirmed a more than fivefold enrichment of various G-protein-coupled receptor signaling pathways (G-protein-coupled adenosine receptor signaling; G-protein-coupled purinergic receptor signaling; G-protein-coupled acetylcholine receptor signaling; G-protein-coupled glutamate receptor signaling). In addition, pathways including cAMP signaling, or including phospholipase C and inositol trisphosphate signaling were more than fivefold enriched, and this was highly significant (Table 1). An endothelin signaling pathway was almost sixfold enriched, and a cGMP signaling pathway was significantly enriched.

**Table 1:** Selected signaling pathways possibly connected to ion transport, metabolic activity, or vasomotor control significantly enriched more than 4.5-fold based on GO enrichment analysis using the GO Ontology database https://doi.org/10.5281/zenodo.6799722, released 2022-07-01. FDR, false discovery rate

GO biological process	Counts	Fold enrichment	Raw *p* value	FDR
G protein-coupled adenosine receptor signaling pathway	10	6.55	8.45E−05	7.66E−04
Positive regulation of inositol trisphosphate biosynthetic process	5	6.55	5.72E−03	3.28E−02
G protein-coupled purinergic receptor signaling pathway	10	6.55	8.45E−05	7.66E−04
Regulation of inositol trisphosphate biosynthetic process	6	6.55	2.42E−03	1.55E−02
Transmembrane receptor protein tyrosine phosphatase signaling pathway	6	6.55	2.42E−03	1.56E−02
G protein-coupled purinergic nucleotide receptor signaling pathway	13	6.09	1.22E−05	1.30E−04
Endothelin receptor signaling pathway	10	5.96	1.42E−04	1.23E−03
cAMP biosynthetic process	9	5.90	3.25E−04	2.61E−03
Regulation of vascular associated smooth muscle contraction	8	5.83	7.45E−04	5.55E−03
Regulation of systemic arterial blood pressure by norepinephrine-epinephrine	8	5.83	7.45E−04	5.54E−03
G protein-coupled opioid receptor signaling pathway	7	5.74	1.72E−03	1.16E−02
Purinergic nucleotide receptor signaling pathway	26	5.50	2.50E−09	4.65E−08
Positive regulation of cGMP-mediated signaling	5	5.46	9.36E−03	4.90E−02
Adenylate cyclase-activating dopamine receptor signaling pathway	8	5.24	1.19E−03	8.33E−03
Positive regulation of protein kinase C signaling	8	5.24	1.19E−03	8.31E−03
G protein-coupled acetylcholine receptor signaling pathway	15	5.17	1.00E−05	1.08E−04
G protein-coupled glutamate receptor signaling pathway	11	5.15	1.60E−04	1.36E−03
cAMP metabolic process	18	5.13	1.43E−06	1.79E−05
Adenylate cyclase-inhibiting G protein-coupled glutamate receptor signaling pathway	7	5.10	2.71E−03	1.70E−02
Activation of phospholipase C activity	23	5.03	6.55E−08	1.01E−06
Adenylate cyclase-activating adrenergic receptor signaling pathway	16	4.99	6.96E−06	7.75E−05

A more detailed analysis revealed expression of a number of receptor proteins involved in blood flow regulation, like atrial natriuretic peptide receptor (anpra), beta-adrenergic receptor kinase (arbk2), and endothelin receptor (ednra; ednrb). At the mRNA transcript level additional transcripts coding for muscarinic acetylcholine receptor m2–m5 have been detected (acm2–acm5), but the proteins have not been found in the proteome.

As already indicated by the GO enrichment analysis, in the transcriptome 114 transcripts connected to G-protein coupled receptor signaling could be detected. Downstream of G-protein coupled receptors 53 transcripts connected to adenylate cyclase signaling were identified. Several guanine nucleotide-binding proteins and inositol-trisphosphate receptor (itpr1, itpr2) were also expressed at the protein level. In addition, calcium calmodulin-dependent protein kinase (kcc2b) was detected in the transcriptome as well as in the proteome.

Growth factor signaling appeared to be of importance in rete mirabile endothelial cells, as 177 transcripts connected to growth factor signaling could be detected, including, for example, epidermal growth factor (eps15), insulin like growth factor (igf1r), hepatocyte growth factor (met), and transforming growth factor (tgbr3; tgfa1; tgfr2), which were also detected in the proteome. In addition, 7 tyrosine-protein kinase receptor proteins and 10 receptor-type tyrosine phosphatase proteins were found. Moreover, tyrosine-kinase receptor proteins connected to angiogenesis have been detected like ephrin type-b receptor (ephb3), tie1 and tie 2 receptor (tie1; tie2), and vascular endothelial growth factor receptor (vegfr2; vegfr4).

Small G-protein signaling also appeared to play an important role, as 67 transcripts connected to rho-protein signal transduction were identified, and 32 transcripts connected to rac protein signal transduction. Numerous small G-proteins (rho; rac; ras) were also present in the proteome.

## Discussion

The number of transcripts coding for a receptor protein or a protein involved in intracellular signaling by far exceeded the number of proteins detected. This at least in part can be explained by the whole genome duplication that occurred in teleost evolution (Glasauer and Neuhauss 2014; Meyer and Van de Peer 2017). Also, a number of genes detected in the transcriptome was not detected in the proteome and vice versa. As repeatedly observed in different species including humans the correlation between protein and mRNA is often modest (Liu et al. 2016; Wang et al. 2019; Schneebauer et al. 2021). There are in fact several phenomena that may contribute to a discrepancy between the transcriptome and the protein expression level, like, for example, stored transcripts, a delay between transcription and translation, post-translational regulation, or post-transcriptional regulation. The main focus of this study, therefore, was on genes present in the transcriptome as well as in the proteome.

A large number of members of the family of G-protein-coupled receptors has been detected in the transcriptome and in the proteome of the rete mirabile of the European eel. The family of G-protein-coupled receptors is a very large and very diverse family of receptor proteins (Lefkowitz 2004; Sanders et al. 2008; Calebiro et al. 2021). It includes adrenergic receptors and muscarinic cholinergic receptors, operating with adenylyl cyclase and protein kinase A (Pierce et al. 2002). The central components of these signaling pathways were detected in the transcriptome as well as in the proteome. Adrenaline has been shown to reduce swimbladder perfusion (Pelster 1994), and the influence of the vagosympathetic trunk on swimbladder blood flow is well documented (Schwerte et al. 1997; Nilsson 2009; Smith and Croll 2011). This suggests that control of blood flow through the swimbladder may be established at the level of the rete mirabile, and blood flow is a crucial parameter determining the rate of gas secretion. In the European eel, an increase in swimbladder perfusion has been shown to result in a proportional increase in the rate of gas secretion into the swimbladder (Pelster and Scheid 1992). Smooth muscle cells at the entrance of the rete could adjust peripheral resistance and thus control swimbladder perfusion. Presence of the endothelin receptor supported the conclusion that blood flow regulation may be possible at the site of the rete mirabile, endothelin being a potent vasoconstrictor (Davenport et al. 2016).

**Fig. 2:**
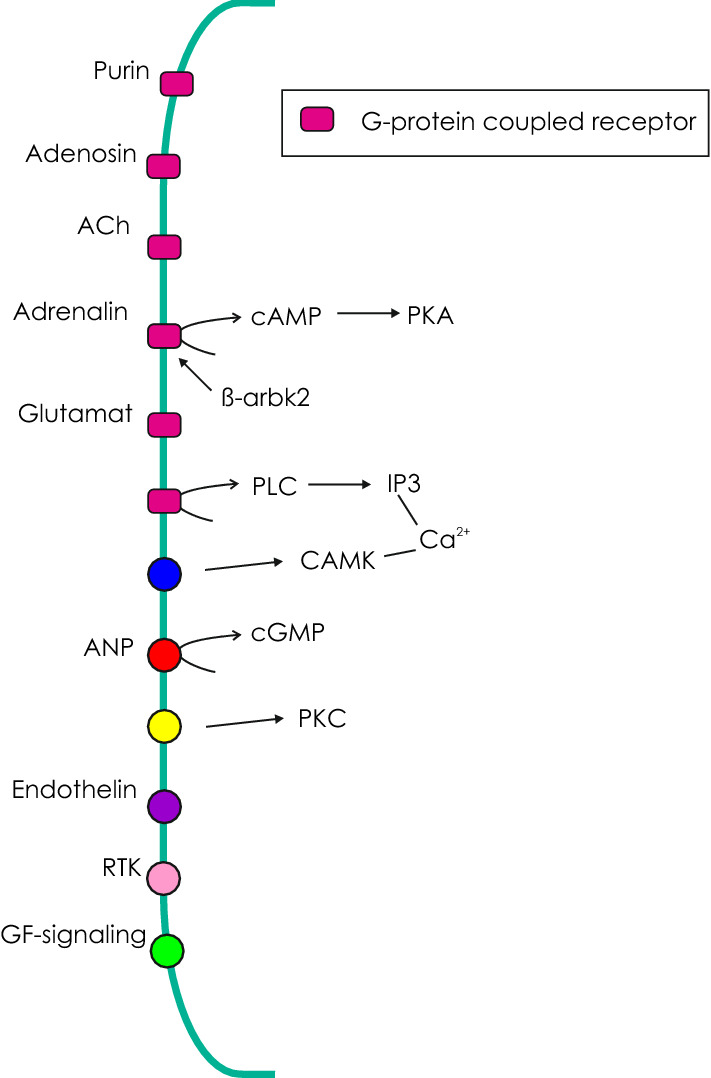
Selected receptor proteins, proteins involved in intracellular signal transduction and assumed signaling pathways in rete mirabile endothelial cells. GF- (growth factor) signaling, several growth factor receptors have been detected including tie1,2; vegf2,4; ephrin receptor. ACh, acetylcholine; ANP, atrial natriuretic peptide; PKA, protein kinase A; PKC, protein kinase C, CAMK, calmodulin dependent protein kinase; ß-arbk2, beta-adrenergic receptor kinase; TRK, receptor tyrosine kinase

Another signaling pathway significantly enriched was the G protein-coupled glutamate receptor signaling pathway, which includes the calcium-sensing receptor (Pierce et al. 2002). The importance of Ca^2+^ as a signaling molecule was also supported by the presence of calcium calmodulin-dependent protein kinase, detected in the proteome, and of the inositol-trisphosphate receptor. Inositol-tris–phosphate is produced by the activity of phospholipase C, activated by a G protein-coupled receptor, and triggers the release of Ca^2+^ from the intracellular stores (like endoplasmic reticulum). Ca^2+^ is essential for activation of smooth muscle cells, which would be consistent with the conclusion that smooth muscle cells at the rete mirabile may modify perfusion resistance and thus control blood flow through the swimbladder, a crucial parameter for swimbladder function. The rate of gas secretion into the swimbladder is correlated to blood flow (Pelster and Scheid 1992), and blood flow influences countercurrent concentration in the rete mirabile (Stevens 2011). In Fig. 2, different G protein-coupled receptors detected are depicted together with possible intracellular signaling molecules, identified in the transcriptome and proteome of the rete.

The presence of atrial natriuretic peptide receptor 1 in the transcriptome and the proteome supported the conclusion that the rete is involved in the control of blood flow through the swimbladder. ANP signaling is involved in the regulation of blood pressure and body fluid volume, and is antagonistic to the renin angiotensin system (Nakagawa et al. 2019). Intracellular signaling includes cGMP signaling, and members of the cGMP mediated signaling pathway were significantly enriched in the transcriptome (Fig. 2).

A remarkable number of growth hormone receptor proteins have been identified, like the receptor tyrosine kinases tie1, tie 2, and vegfr2 and vegfr4, potent angiogenic growth factor receptors (Simons et al. 2016; Akwii et al. 2019). In addition, ephrin type-b receptor, involved in morphogenesis and cell differentiation (Wilkinson 2014), but also in angiogenesis and vasculogenesis (Zhang and Hughes 2006), has been detected (Fig. 2). Schneebauer et al. compared yellow and silver eels and found only a small number of genes differentially expressed in the rete, so that the two different developmental stages were combined in their analysis focusing on transport protein expression in the rete (Schneebauer et al. 2021). Studies on the American eel *Anguilla rostrata*, however, revealed an increase in rete length (Kleckner and Krueger 1981), suggesting that angiogenesis should be activated in silver eels. Elongation of the rete increases surface area available for countercurrent exchange and, therefore, significantly improves the capacity for countercurrent multiplication (Kobayashi et al. 1989b). The transcript of angiopoietin-related protein 4 was indeed elevated in the expression level in silver eels, but the according protein was not detected in the proteome (Schneebauer et al. 2021; this study). Based on the silvering and ocular index, the silver eels used by Schneebauer et al. were far beyond the threshold for the transition from yellow to silver eels (Schneebauer et al. 2021). Therefore, it appears possible that elongation of the rete was close to complete and elevated angiogenic signaling to stimulate further elongation of the rete was no longer necessary.

With respect to membrane transport proteins V-ATPase and aquaporin have been shown to contribute to acid back-diffusion in the rete, significantly enhancing the countercurrent concentration of oxygen and CO_2_, whereas monocarboxylate carriers allow for a back-diffusion of lactate, facilitating countercurrent concentration of inert gases by supporting the salting out effect (Kobayashi et al. 1989b, 1990; Schneebauer et al. 2021). V-ATPase and also aquaporin are well known to be introduced into cell membranes by trafficking of vesicles between cytoplasm and cell membranes. Both transport proteins are inserted into the cell membrane following hormonal activation of G-protein-coupled receptors, followed by activation of adenylyl cyclase and protein kinase A (Brown et al. 1998; Nedvetsky et al. 2009; Mcguire et al. 2017; Collins and Forgac 2020). As already discussed, the G-protein-coupled signaling pathway was significantly enriched and crucial components of these signaling pathways were expressed in rete tissue. It, therefore, appears quite possible that regulated insertion of aquaporin and V-ATPase support back-diffusion and countercurrent concentration in the rete. The same appears to be true for monocarboxylate transporters. cAMP dependent trafficking of monocarboxylate carriers has been shown in rat brain endothelial cells (Smith et al. 2012; Uhernik et al. 2014). The anxiliary proteins basigin and embigin have been shown to be involved in monocarboxylate trafficking (Nakai et al. 2006; Felmlee et al. 2020), and basigin has been detected in the transcriptome, but not in the proteome. The proteome and transcriptome data therefore reveal that the rete mirabile is equipped with the required signaling pathways to allow for a regulated insertion of transport proteins into rete membranes. The number of transport proteins affects back-diffusion of metabolites, and thus influences countercurrent multiplication achieved in the rete.

Taken together the data suggest that the rete mirabile is equipped with the capacity to regulate blood flow through the rete, and to regulate back-diffusion of metabolites in the rete. Both parameters are crucial components for the effectiveness of the rete as a countercurrent multiplier. The rete, therefore, appears to be a crucial component significantly influencing the secretory activity of the swimbladder.

## Supplementary Information

Below is the link to the electronic supplementary material.

Supplementary file1 (XLSX 38 KB)

## Data Availability

Data are available under: transcriptome: GSE172092, https://www.ncbi.nlm.nih.gov/geo/query/acc.cgi?acc=GSE172092 and proteome data (accession number PXD025435, https://www.ebi.ac.uk/pride/archive/projects/PXD025435).

## References

[CR1] Akwii RG, Sajib MS, Zahra FT, Mikelis CM (2019). Role of angiopoietin-2 in vascular physiology and pathophysiology. Cells.

[CR2] Berenbrink M, Koldkjaer P, Kepp O, Cossins AR (2005). Evolution of oxygen secretion in fishes and the emergence of a complex physiological system. Science.

[CR3] Brown D, Katsura T, Gustafson CE (1998). Cellular mechanisms of aquaporin trafficking. Am J Physiol Physiol.

[CR4] Calebiro D, Koszegi Z, Lanoiselée Y, Miljus T, O’Brien S (2021). G protein-coupled receptor-G protein interactions: a single-molecule perspective. Physiol Rev.

[CR5] Collins MP, Forgac M (2020). Regulation and function of V-ATPases in physiology and disease. Biochim Biophys Acta Biomembr.

[CR6] Davenport AP, Hyndman KA, Dhaun N, Southan C, Kohan D, Pollock J, Pollock D, Webb D, Maguire J (2016). Endothelin. Pharmacol Rev.

[CR7] Dickson KA, Graham JB (2004). Evolution and consequences of endothermy in fishes. Physiol Biochem Zool.

[CR8] Dorn E (1961). Über den Feinbau der Schwimmblase von *Anguilla vulgaris* L. Licht- und Elektronenmikroskopische Untersuchungen. Zeitschrift Für Zellforsch.

[CR9] Fänge R (1983). Gas exchange in fish swim bladder. Rev Physiol Pharmacol.

[CR10] Felmlee MA, Jones RS, Rodriguez-Cruz V (2020). Monocarboxylate transporters (SLC16): function, regulation, and role in health and disease. Pharmacol Rev.

[CR11] Glasauer SMK, Neuhauss SCF (2014). Whole-genome duplication in teleost fishes and its evolutionary consequences. Mol Genet Genom.

[CR12] Howe K, Clark MD, Torroja CF (2013). The zebrafish reference genome sequence and its relationship to the human genome. Nature.

[CR13] Jessen C (2001). Selective brain cooling in mammals and birds. Jpn J Physiol.

[CR14] Kleckner RC, Krueger WH (1981). Changes in swimbladder retial morphology in *Anguilla rostrata* during premigration metamorphosis. J Fish Biol.

[CR15] Kobayashi H, Pelster B, Scheid P (1989). Water and lactate movement in the swimbladder of the eel, *Anguilla anguilla*. Respir Physiol.

[CR16] Kobayashi H, Pelster B, Scheid P (1989). Solute back-diffusion raises the gas concentrating efficiency in counter-current flow. Respir Physiol.

[CR17] Kobayashi H, Pelster B, Scheid P (1990). CO_2_ back-diffusion in the rete aids O_2_ secretion in the swimbladder of the eel. Respir Physiol.

[CR18] Krogh A (1929). The anatomy and physiology of capillaries.

[CR19] Kuhn W, Ramel A, Kuhn HJ, Marti E (1963). The filling mechanism of the swimbladder. Generation of high gas pressures through hairpin countercurrent multiplication. Experientia.

[CR20] Lefkowitz RJ (2004). Historical review: a brief history and personal retrospective of seven-transmembrane receptors. Trends Pharmacol Sci.

[CR21] Liu Y, Beyer A, Aebersold R (2016). On the dependency of cellular protein levels on mRNA abundance. Cell.

[CR22] Marshall NB (1960). Swimbladder structure of deep-sea fishes in relation to their systematics and biology. DiscoverReports.

[CR23] McCafferty DJ, Gilbert C, Thierry A-M, Currie J, Le Maho Y, Ancel A (2013). Emperor penguin body surfaces cool below air temperature. Biol Lett.

[CR24] Mcguire C, Stransky L, Cotter K, Forgac M (2017). Regulation of V-ATPase activity. Front Biosci.

[CR25] Meyer A, Van de Peer Y (2017). From 2R to 3R: evidence for a fish-specific genome duplication (FSGD). BioEssays.

[CR26] Nakagawa Y, Nishikimi T, Kuwahara K (2019). Atrial and brain natriuretic peptides: hormones secreted from the heart. Peptides.

[CR27] Nakai M, Chen L, Nowak RA (2006). Tissue distribution of basigin and monocarboxylate transporter 1 in the adult male mouse: a study using the wild-type and basigin gene knockout mice. Anat Rec Part A Discov Mol Cell Evol Biol.

[CR28] Nedvetsky PI, Tamma G, Beulshausen S, Valenti G, Rosenthal W, Klussmann E, Beitz E (2009). Regulation of aquaporin-2 trafficking. Aquaporins. Handbook of experimental pharmacology.

[CR29] Nilsson S (2009). Nervous control of fish swimbladders. Acta Histochem.

[CR30] Pelster B (1994). Adrenergic control of swimbladder perfusion in the European eel *Anguilla anguilla*. J Exp Biol.

[CR31] Pelster B (2001). The generation of hyperbaric oxygen tensions in fish. Physiology.

[CR32] Pelster B, Randall DJ, Farrel AP (1997). Buoyancy at depth. Fish physiology.

[CR33] Pelster B (2023). Swimbladder function in the European eel *Anguilla anguilla*. Fishes.

[CR34] Pelster B, Scheid P (1992). The influence of gas gland metabolism and blood flow on gas deposition into the swimbladder of the European eel *Anguilla anguilla*. J Exp Biol.

[CR35] Pierce KL, Premont RT, Lefkowitz RJ (2002). Seven-transmembrane receptors. Nat Rev Mol Cell Biol.

[CR36] Sanders RD, Brian D, Maze M, Schüttler J, Schwilden H (2008). G-protein-coupled receptors. Moderne anesthetics. Handbook of experimental pharmacology.

[CR37] Schneebauer G, Drechsel V, Dirks R, Faserl K, Sarg B, Pelster B (2021). Expression of transport proteins in the rete mirabile of European silver and yellow eel. BMC Genom.

[CR38] Scholander PF, Schevill WE (1955). Counter-current vascular heat exchange in the fins of whales. J Appl Physiol.

[CR39] Schwerte T, Axelsson M, Nilsson S, Pelster B (1997). Effects of vagal stimulation on swimbladder blood flow in the european eel *Anguilla anguilla*. J Exp Biol.

[CR40] Simons M, Gordon E, Claesson-Welsh L (2016). Mechanisms and regulation of endothelial VEGF receptor signalling. Nat Rev Mol Cell Biol.

[CR41] Smith FM, Croll RP (2011). Autonomic control of the swimbladder. Auton Neurosci.

[CR42] Smith JP, Uhernik AL, Li L, Liu Z, Drewes LR (2012). Regulation of Mct1 by cAMP-dependent internalization in rat brain endothelial cells. Brain Res.

[CR43] Steen JB (1963). The physiology of the swimbladder in the eel *Anguilla vulgaris*. III. The mechanism of gas secretion. Acta Physiol Scand.

[CR44] Stevens ED (2011) Design and physiology of arteries and veins. The Retia. In: Encyclopedia of Fish Physiology. Elsevier, pp 1119–1131

[CR45] Strauss WM, Hetem RS, Mitchell D, Maloney SK, O'Brien HD, Meyer LCR, Fuller A (2017). Body water conservation through selective brain cooling by the carotid rete: a physiological feature for surviving climate change?. Conserv Physiol.

[CR46] Stray-Pedersen S, Nicolaysen A (1975). Qualitative and quantitative studies of the capillary structure in the rete mirabile of the eel, *Anguilla vulgaris* L. Acta Physiol Scand.

[CR47] Uhernik AL, Li L, LaVoy N, Velasquez MJ, Smith JP (2014). Regulation of monocarboxylic acid transporter-1 by cAMP dependent vesicular trafficking in brain microvascular endothelial cells. PLoS ONE.

[CR48] Wagner RC, Froehlich R, Hossler FE, Andrews SB (1987). Ultrastructure of capillaries in the red body (rete mirabile) of the eel swim bladder. Microvasc Res.

[CR49] Wang D, Eraslan B, Wieland T (2019). A deep proteome and transcriptome abundance atlas of 29 healthy human tissues. Mol Syst Biol.

[CR50] Waser W (2011) Transport and exchange of respiratory gases in the blood. Root effect: root effect definition, functional role in oxygen delivery to the eye and swimbladder. In: Encyclopedia of fish physiology. Elsevier, pp 929–934

[CR51] Wilkinson DG (2014). Regulation of cell differentiation by Eph receptor and ephrin signaling. Cell Adh Migr.

[CR52] Zhang J, Hughes S (2006). Role of the ephrin and Eph receptor tyrosine kinase families in angiogenesis and development of the cardiovascular system. J Pathol.

[CR53] Zwerger P, Nimeth K, Würtz J, Salvenmoser W, Pelster B (2002). Development of the swimbladder in the European eel (*Anguilla anguilla*). Cell Tissue Res.

